# Application of a deep learning-based system for eyelid margin signs identification training and testing in dry eye disease education

**DOI:** 10.3389/fmed.2026.1896446

**Published:** 2026-07-31

**Authors:** Ningkai Tang, Zhixin Duan, Yuexin Wang

**Affiliations:** 1 Department of Ophthalmology, Peking University Third Hospital, Beijing, China; 2 Peking University Health Science Center, Beijing, China

**Keywords:** artificial intelligence, deep learning, dry eye disease, eyelid margin, medical education

## Abstract

**Background:**

Dry eye disease (DED) is a common ocular surface disease, and identifying abnormal eyelid margin signs is essential for clinical diagnosis. However, traditional teaching methods lack effective feedback and often lead to low learning efficiency. This study aimed to develop an interactive teaching platform based on a deep learning model for eyelid margin signs and to evaluate its effectiveness.

**Methods:**

This randomized controlled trial included 40 medical students from the Peking University Health Science Center. Participants were randomly assigned to a conventional learning group (*n* = 19) or a platform learning group (*n* = 21). After baseline training, the platform group practiced using the AI-assisted system with real-time feedback, while the conventional group reviewed video lectures. Two weeks later, all students took a timed final exam evaluating eight core abnormal eyelid margin signs.

**Results:**

The platform learning group achieved a significantly higher total score than the conventional learning group (56.95 ± 5.95 vs. 53.37 ± 4.32, *p* = 0.035). Specifically, the platform group showed a significantly higher accuracy rate in identifying meibomian gland orifice (MGO) plugging (77.6% vs. 67.4%, *p* = 0.021). Subgroup analysis revealed that the platform significantly improved the accuracy of rounding of posterior lid margin for undergraduate students (82.1% vs. 69.1%, *p* = 0.016), and the accuracy of MGO plugging for graduate students (85.7% vs. 67.5%, *p* = 0.009).

**Conclusion:**

The deep learning-integrated teaching platform may improve students’ overall accuracy in identifying abnormal eyelid margin signs, particularly MGO plugging. This platform may serve as a useful supplement to traditional clinical ophthalmology education and may help improve the efficiency of learning eyelid margin assessment.

## 1 Introduction

In recent years, the incidence of dry eye disease (DED) has increased significantly, possibly due to factors like the widespread use of mobile devices and worsening environmental pollution (1). It has become a common chronic ocular surface disease that severely impacts people’s quality of life and work efficiency (1). The core characteristic of DED is the loss of tear film homeostasis. The human tear film consists of three main layers: mucin, aqueous, and lipid. The outermost lipid layer is primarily secreted by the meibomian glands. The orifices of these glands are located anterior to the mucocutaneous junction (MCJ) of the eyelid margin. This anatomical position allows the meibum to be secreted and spread across the tear film surface, which maintains tear film stability and delays tear evaporation (1–4). Therefore, the meibomian glands and the eyelid margin are essential components of the tear film functional unit. Abnormal signs of the eyelid margin, including round margin, notching, vascular dilation, orifice obstruction and displacement, and MCJ displacement, are essential signs in various types of DED (5). When combined with ocular symptoms, these signs help establish a classification diagnosis of DED that guides the treatment (6–8).

Currently, the primary teaching and training method for abnormal eyelid margin signs for medical students and junior doctors involves representation and explanation of signs from anterior segment photographs and in-field slit lamp that show typical features by senior doctors. However, this traditional method has several limitations (9, 10). First, the evaluation relies heavily on the personal experience of the doctors, which may lead to subjective bias. Second, the anatomy of the eyelid margin is complex, and the educational content involves nearly 10 abnormalities to teach. The traditional teaching method separates training and testing and lacks effective feedback. It results in a long learning curve, quick memory loss, and low overall learning efficiency.

Recently, artificial intelligence (AI) has been widely applied in the diagnosis and evaluation of dry eye disease. Several reviews and studies show that AI models can automatically evaluate tear film parameters and meibomian gland conditions from clinical images (11, 12). These AI models can achieve a high diagnostic accuracy of 84–92% for dry eye disease, demonstrating clinical effectiveness that is comparable to expert ophthalmologists (13–16). In our previous research, a deep learning model was developed to identify abnormal eyelid margin signs from anterior segment photography. These signs include rounding of the posterior lid margin, lid margin irregularity, lid margin vascularization, meibomian gland orifice (MGO) plugging, MGO retroplacement and anteroplacement, and MCJ anteroplacement and retroplacement. The model achieved high accuracy in detecting these abnormalities (5). The advantages of deep learning models, such as their speed and accuracy, could be integrated into a teaching platform to promote learning efficiency. However, this application remains to be explored.

Thus, the present study aims to develop an interactive DED teaching platform based on the previous deep learning model for lid margin sign teaching and testing. Furthermore, the efficiency of the platform was evaluated with a randomized controlled study that compared the examination results between the platform and traditional teaching methods.

## 2 Materials and methods

### 2.1 Participants and study design

This study was conducted as a randomized controlled trial to evaluate an online teaching platform for dry eye disease. A total of 40 students from the Peking University Health Science Center were recruited as participants. The inclusion criteria included 3rd- to 5th-year undergraduate students who had not systematically studied ophthalmology, and graduate students who had completed basic ophthalmology but had not taken a specialized dry eye course. Students who did not meet these specific educational backgrounds were excluded from the study. Written informed consent was obtained from all participants before the experiment.

### 2.2 Randomization and masking

The included participants were randomly assigned to two groups: a conventional learning group (*n* = 19) and a platform learning group (*n* = 21). The assignment was performed using a stratified randomization method. Specifically, the randomization process was stratified based on the participants’ gender and educational level to ensure a balanced distribution. The allocation sequence was concealed in sequentially numbered. Due to the nature of the educational intervention, blinding of the participants to their group allocation was not possible and the outcome assessor was blinded to group allocation.

### 2.3 Platform development

The backend of the online teaching platform was built using the Spring Boot framework, and the frontend was developed with Vue (17). FastAPI was implemented to manage communication with the deep learning module. The core function of the platform is the embedded artificial intelligence model. When anterior segment photographs of the eyelid margins are input into the system, the model automatically identifies and annotates the abnormal eyelid margin signs. Specifically, the deep learning model was constructed based on VGGNet-13 with batch normalization layers and was trained on 832 anterior segment photographs from DED patients (5). The model demonstrated high performance in identify lid margin abnormalities, including rounding of posterior lid margin, lid margin irregularity, lid margin vascularization, MGO plugging, MGO anteroplacement, MGO retroplacement, MCJ anteroplacement, and MCJ retroplacement. After annotation by the model, these images are automatically added to the question bank. The correctness of the annotation was further validated by experienced ophthalmologists.

The platform includes a user management module that separates system permissions for different user identities, specifically teachers and students. Users with teacher permissions can generate eyelid margin training exercises and exam question sets. In addition, teachers can view the students’ practice records and exam results. On the other hand, students can use the platform to conduct autonomous training. During the practice process, the system calls the deep learning model to provide objective, real-time feedback. Students can also participate in the exams published by the teachers. To prevent data leakage, all images in the question bank could not be linked to individual patients. The practice and exam images were partially sourced from clinical cohort and might overlap the training and validation datasets of the deep learning model.

### 2.4 Procedure

A participant flow diagram is provided as Supplementary Figure S1. First, all 40 students attended a baseline training. This training included a 40-min lecture on dry eye disease and eyelid margin anatomy, followed by a 30-min session for analyzing typical images of abnormal eyelid margin signs. A senior doctor delivered all these baseline courses.

Over the following week, the students completed different training tasks based on their assigned groups. The platform learning group used the developed teaching system for a 30-min free practice session. During this session, the system provided real-time feedback and corrections for each diagnosis. At the same time, the conventional learning group spent 30 min reviewing the material by watching recorded lecture videos.

Two weeks after the baseline training, all students took a 30-min timed final exam on the teaching platform. The exam evaluated eight core abnormal eyelid margin signs: rounding of posterior lid margin, lid margin irregularity, lid margin vascularization, MGO plugging, MGO anteroplacement, MGO retroplacement, MCJ anteroplacement, and MCJ retroplacement.

The final exam consisted of 10 image-based questions. These questions used 10 standardized anterior segment photographs of eyelid margins that showed one or more abnormal signs. For each photograph, students were required to make binary (yes/no) judgments regarding the presence of the eight specific signs. Therefore, each question was worth 8 points, making a total possible score of 80 points. Each of the eight signs was evaluated 10 times across the exam, accounting for 10 points per sign. The final total score was calculated as the sum of these eight individual sign scores.

### 2.5 Statistical analysis

All statistical analyses were performed using SPSS software (version 26.0). An *a priori* power analysis was not performed due to the exploratory nature of this educational study. This study evaluated the overall accuracy in identifying the eight key abnormal eyelid margin signs. The accuracy rate for each abnormal sign was calculated by dividing the total number of correct judgments by the total number of judgments made for that specific sign. Test scores and accuracy rates were compared between the conventional learning group and the platform learning group. Additionally, a subgroup analysis was conducted based on the students’ prior medical knowledge, specifically comparing undergraduates and graduate students. Categorical variables, including gender distributions, were analyzed using the Chi-square test. For continuous variables, the independent samples *t*-test was utilized if the data followed a normal distribution. In contrast, non-parametric tests were applied for non-normally distributed data. Given that accuracy rates were calculated from multiple judgments per student and image, these observations are not strictly independent. A mixed-effects logistic regression models with participant-level and image-level random intercepts was applied to compare the accuracy rate between two groups A *p*-value < 0.05 was considered statistically significant.

## 3 Results

### 3.1 Baseline characteristics of participants

Ultimately, 40 valid participants completed the prospective experiment and were included in the statistical analysis: 19 in the conventional learning group and 21 in the platform learning group. The two groups demonstrated high comparability, with no significant differences in gender distribution (males: 47.4% vs. 47.6%, *p* = 0.987) or educational background (undergraduates: 57.9% vs. 66.7%, *p* = 0.567).

### 3.2 Overall accuracy and error patterns

Table 1 presents the overall accuracy, false-positive error, and false-negative error rates for each abnormal eyelid margin sign. The identification accuracy was relatively high for MCJ anteroplacement (76.8%) and MGO anteroplacement (75.8%). Other signs with accuracy rates above 70% included rounding of posterior lid margin (73.0%) and MGO plugging (72.8%). The accuracy rates for MCJ retroplacement and lid margin irregularity were 69.5 and 65.0%, respectively. In contrast, the accuracy rates for MGO retroplacement (60.5%) and lid margin vascularization (59.5%) were notably lower.

**Table 1:** Overall accuracy and error rates for each abnormal eyelid margin sign.

Lid margin sign	Accuracy rates	False-positive error^#^	False-negative error^*^
Rounding of posterior lid margin	73.0%	21.3%	5.8%
Lid margin irregularity	65.0%	24.0%	11.0%
Lid margin vascularization	59.5%	20.5%	20.0%
MGO plugging	72.8%	11.3%	16.0%
MGO retroplacement	60.5%	15.3%	24.3%
MGO anteroplacement	75.8%	14.0%	10.3%
MCJ anteroplacement	76.8%	17.5%	5.8%
MCJ retroplacement	69.5%	15.5%	15.0%

#The abnormal sign was absent in the image but incorrectly judged as present by the student.

^*^The abnormal sign was present in the image but incorrectly missed by the student.

MCJ, mucocutaneous junction; MGO, meibomian gland orifice.

Regarding error patterns, several signs were prone to false-positive judgments, meaning the abnormal sign was absent in the image but incorrectly judged as present by the student. The highest false-positive error rates were observed in lid margin irregularity (24.0%), rounding of posterior lid margin (21.3%), and lid margin vascularization (20.5%). The false-positive rates for the remaining signs were 17.5% for MCJ anteroplacement, 15.5% for MCJ retroplacement, 15.3% for MGO retroplacement, 14.0% for MGO anteroplacement, and 11.3% for MGO plugging. Conversely, some signs showed a higher tendency for false-negative errors, where the sign was present in the image but incorrectly missed during diagnosis. MGO retroplacement (24.3%) and lid margin vascularization (20.0%) showed the highest false-negative rates. They were followed by MGO plugging (16.0%), MCJ retroplacement (15.0%), lid margin irregularity (11.0%), and MGO anteroplacement (10.3%). The lowest false-negative error rates were both 5.8%, found in the rounding of posterior lid margin and the MCJ anteroplacement.

### 3.3 Comparison of learning outcomes between groups

Table 2 shows the itemized scores and total scores for the eight abnormal eyelid margin signs between the Conventional Learning Group (*n* = 19) and the Platform Learning Group (*n* = 21). The Platform Learning Group achieved a significantly higher total score compared to the Conventional Learning Group (56.95 ± 5.95 vs. 53.37 ± 4.32, *p* = 0.035). For the specific signs, the score for MGO plugging was significantly higher in the platform group (7.76 ± 2.30) than in the conventional group (6.74 ± 1.15, *p* = 0.020), and the platform group demonstrated a trend toward better performance in identifying the MCJ anteroplacement (8.00 ± 0.89 vs. 7.26 ± 1.33, *p* = 0.054). However, there were no significant differences between the two groups in the scores of the other signs. These included rounding of posterior lid margin (*p* = 0.205), lid margin irregularity (*p* = 0.399), lid margin vascularization (*p* = 0.748), MGO retroplacement (*p* = 0.533), MGO anteroplacement (*p* = 0.361), and MCJ retroplacement (*p* = 0.957).

**Table 2:** Comparison of itemized scores for each abnormal eyelid margin sign and total scores between the two groups*.

Lid margin sign	Conventional learning group (*n* = 19)	Platform learning group (*n* = 21)	*p* ^#^
Rounding of posterior lid margin	7.00 ± 1.76	7.57 ± 2.29	0.205
Lid margin irregularity	6.26 ± 1.63	6.71 ± 1.71	0.399
Lid margin vascularization	6.16 ± 1.86	5.76 ± 2.14	0.748
MGO plugging	6.74 ± 1.15	7.76 ± 2.30	**0.020**
MGO retroplacement	5.84 ± 1.83	6.24 ± 2.12	0.533
MGO anteroplacement	7.21 ± 1.96	7.90 ± 1.67	0.361
MCJ anteroplacement	7.26 ± 1.33	8.00 ± 0.89	0.054
MCJ retroplacement	6.89 ± 1.94	7.00 ± 1.55	0.957
Total Scores	53.37 ± 4.32	56.95 ± 5.95	**0.035**

^*^The item score for a specific abnormal sign was defined as the number of correct judgments across the 10 questions. The total score was calculated as the sum of the scores for all eight signs.

^#^The independent samples *t*-test was used for normally distributed data, whereas non-parametric tests were applied to non-normally distributed data.

MCJ, mucocutaneous junction; MGO, meibomian gland orifice.

The bold values mean significant difference.

Table 3 presents the comparison of accuracy rates for identifying each abnormal sign between the two groups. Consistent with the score results, the Platform Learning Group had a significantly higher accuracy rate in identifying MGO plugging compared to the Conventional Learning Group (77.6% vs. 67.4%, *p* = 0.049). For the other signs, although the platform group showed generally higher accuracy rates in most items, these differences did not reach statistical significance. Specifically, no significant differences were found for rounding of posterior lid margin (75.7% vs. 70.0%, *p* = 0.199), lid margin irregularity (67.1% vs. 62.6%, *p* = 0.345), MGO retroplacement (62.4% vs. 58.4%, *p* = 0.418), MGO anteroplacement (79.0% vs. 72.1%, *p* = 0.106), MCJ anteroplacement (80.0% vs. 72.6%, *p* = 0.083), and MCJ retroplacement (70.0% vs. 68.9%, *p* = 0.819). Notably, the accuracy rate for identifying lid margin vascularization was slightly lower in the platform group, but this difference was also not significant (57.6% vs. 61.6%, *p* = 0.420).

**Table 3:** Comparison of accuracy rates for each abnormal eyelid margin sign between the two groups*.

Lid margin sign	Conventional learning group	Platform learning group	*p* ^#^
Rounding of posterior lid margin, times (%)	133 (70.0%)	159 (75.7%)	0.363
Lid margin irregularity, times (%)	119 (62.6%)	141 (67.1%)	0.349
Lid margin vascularization, times (%)	117 (61.6%)	121 (57.6%)	0.574
MGO Plugging, times (%)	128 (67.4%)	163 (77.6%)	**0.049**
MGO retroplacement, times (%)	111 (58.4%)	131 (62.4%)	0.541
MGO anteroplacement, times (%)	137 (72.1%)	166 (79.0%)	0.265
MCJ anteroplacement, times (%)	138 (72.6%)	168 (80.0%)	0.096
MCJ retroplacement, times (%)	131 (68.9%)	147 (70.0%)	0.815

^*^Calculated as the number of correct judgments for a specific sign divided by the total number of judgments for that sign.

^#^Analyzed using the Chi-square test.

MCJ, mucocutaneous junction; MGO, meibomian gland orifice.

The bold values mean significant difference.

### 3.4 Subgroup analysis based on educational level

To investigate the influence of prior medical knowledge on the effectiveness of the new teaching modality, a subgroup analysis was performed based on the students’ educational levels.

Table 4 presents the subgroup analysis of itemized and total scores based on educational level. For undergraduate students, there were no significant differences between the Conventional Learning Group (*n* = 11) and the Platform Learning Group (*n* = 14) in the total scores (53.18 ± 5.12 vs. 57.21 ± 5.71, *p* = 0.080) or any of the eight itemized scores (all *p* > 0.05). These specific items included rounding of posterior lid margin (*p* = 0.075), lid margin irregularity (*p* = 0.715), lid margin vascularization (*p* = 0.428), MGO plugging (*p* = 0.464), MGO retroplacement (*p* = 0.936), MGO anteroplacement (*p* = 0.139), MCJ anteroplacement (*p* = 0.218), and MCJ retroplacement (*p* = 0.188). For graduate students (Conventional *n* = 8, Platform *n* = 7), the Platform Learning Group scored significantly higher in MGO plugging compared to the Conventional Learning Group (8.57 ± 1.62 vs. 6.75 ± 0.89, *p* = 0.016). However, no significant differences were found between the two graduate groups in the total scores (*p* = 0.319) and the scores of the other seven signs (all *p* > 0.05).

**Table 4:** Subgroup analysis of itemized and total scores based on educational level*.

Lid margin sign	Conventional learning group	Platform learning group	*p* ^#^
Undergraduate students			
*N*	11	14	/
Rounding of posterior lid margin	6.91 ± 1.87	8.21 ± 1.85	0.075
Lid margin irregularity	6.18 ± 1.78	6.43 ± 1.55	0.715
Lid margin vascularization	6.18 ± 1.94	5.50 ± 2.21	0.428
MGO Plugging	6.73 ± 1.35	7.36 ± 2.53	0.464
MGO retroplacement	6.09 ± 2.21	6.14 ± 2.28	0.936
MGO anteroplacement	7.45 ± 1.57	8.29 ± 1.14	0.139
MCJ anteroplacement	7.45 ± 1.44	8.07 ± 1.00	0.218
MCJ retroplacement	6.18 ± 2.18	7.21 ± 1.63	0.188
Total Scores	53.18 ± 5.12	57.21 ± 5.71	0.080
Graduate students			
*N*	8	7	/
Rounding of posterior lid margin	7.13 ± 1.73	6.29 ± 2.69	0.479
Lid margin irregularity	6.38 ± 1.51	7.29 ± 1.98	0.330
Lid margin vascularization	6.13 ± 1.89	6.29 ± 2.06	0.877
MGO Plugging	6.75 ± 0.89	8.57 ± 1.62	**0.016**
MGO retroplacement	5.50 ± 1.20	6.43 ± 1.90	0.271
MGO anteroplacement	6.88 ± 2.47	7.14 ± 2.34	0.867
MCJ anteroplacement	7.00 ± 1.20	7.86 ± 0.69	0.232
MCJ retroplacement	7.88 ± 0.99	6.57 ± 1.40	0.054
Total Scores	53.63 ± 3.25	56.43 ± 6.85	0.319

^*^The item score for a specific abnormal sign was defined as the number of correct judgments across the 10 questions. The total score was calculated as the sum of the scores for all eight signs.

^#^The independent samples *t*-test was used for normally distributed data, whereas non-parametric tests were applied to non-normally distributed data.

MCJ, mucocutaneous junction; MGO, meibomian gland orifice.

The bold values mean significant difference.

Table 5 shows the subgroup analysis of accuracy rates for identifying each sign based on educational level. Among undergraduate students, the Platform Learning Group demonstrated a significantly higher accuracy rate for rounding of posterior lid margin than the Conventional Learning Group (82.1% vs. 69.1%, *p* = 0.016). There were no significant differences between the undergraduate groups in the accuracy rates of the remaining seven signs, including lid margin irregularity (*p* = 0.688), lid margin vascularization (*p* = 0.278), MGO plugging (*p* = 0.277), MGO retroplacement (*p* = 0.933), MGO anteroplacement (*p* = 0.108), MCJ anteroplacement (*p* = 0.242), and MCJ retroplacement (*p* = 0.083). For graduate students, the accuracy rate for identifying MGO plugging was significantly higher in the platform group than in the conventional group (85.7% vs. 67.5%, *p* = 0.009). Similar to the undergraduate results, no significant differences were observed between the two graduate groups in the accuracy rates of the other seven signs (all *p* > 0.05).

**Table 5:** Subgroup analysis of accuracy rates based on educational level*.

Lid margin sign	Conventional learning group	Platform learning group	*p* ^#^
Undergraduate students			
Rounding of posterior lid margin, times (%)	76 (69.1%)	115 (82.1%)	**0.016**
Lid margin irregularity, times (%)	68 (61.8%)	90 (64.3%)	0.688
Lid margin vascularization, times (%)	68 (61.8%)	77 (55.0%)	0.278
MGO Plugging, times (%)	74 (67.3%)	103 (73.6%)	0.277
MGO retroplacement, times (%)	67 (60.9%)	86 (61.4%)	0.933
MGO anteroplacement, times (%)	82 (74.5%)	116 (82.9%)	0.108
MCJ anteroplacement, times (%)	82 (74.5%)	113 (80.7%)	0.242
MCJ retroplacement, times (%)	68 (61.8%)	101 (72.1%)	0.083
Graduate students			
Rounding of posterior lid margin, times (%)	57 (71.3%)	44 (62.9%)	0.274
Lid margin irregularity, times (%)	51 (63.7%)	51 (72.9%)	0.233
Lid margin vascularization, times (%)	49 (61.3%)	44 (62.9%)	0.840
MGO Plugging, times (%)	54 (67.5%)	60 (85.7%)	**0.009**
MGO retroplacement, times (%)	44 (55.0%)	45 (64.3%)	0.248
MGO anteroplacement, times (%)	55 (68.8%)	50 (71.4%)	0.721
MCJ anteroplacement, times (%)	56 (70.0%)	55 (78.6%)	0.232
MCJ retroplacement, times (%)	63 (78.8%)	46 (65.7%)	0.074

^*^Calculated as the number of correct judgments for a specific sign divided by the total number of judgments for that sign.

^#^Analyzed using the Chi-square test.

MCJ, mucocutaneous junction; MGO, meibomian gland orifice.

The bold values mean significant difference.

## 4 Discussion

Dry eye disease is a common ocular surface disorder, and observing eyelid margin signs is necessary for clinical diagnosis. However, traditional teaching methods mainly use textbooks and lectures, which makes it hard for students to recognize complex morphological details. This study successfully integrated cutting-edge deep learning technology with clinical medical education to develop and validate an innovative online teaching system for identifying eyelid margin signs in dry eye disease. Our findings indicate that students using this platform achieved better overall performance compared to traditional learning methods.

Regarding the overall test results, the identification accuracy was relatively high for MCJ anteroplacement and MGO anteroplacement. In contrast, the accuracy rates for MGO retroplacement and lid margin vascularization were the lowest. A possible reason is that MCJ and MGO anteroplacements involve obvious structural changes, whereas MGO retroplacement and fine blood vessels are often hard to see clearly in two-dimensional photographs. Furthermore, students frequently made false-positive errors for lid margin irregularity and rounding of posterior lid margin. This might occur because beginners tend to mistake normal anatomical variations for pathological changes. On the other hand, MGO retroplacement and lid margin vascularization showed high false-negative rates. Detecting microscopic features like telangiectasia usually requires high-magnification slit-lamp biomicroscopy and extensive clinical experience, making them very easy to miss in standard images for non-experts (18).

When comparing the two groups, the platform learning group achieved significantly higher overall scores than the conventional learning group. Specifically, the platform group performed much better in identifying MGO plugging. While existing literature shows that AI-based educational systems can enhance learning through autonomous tutoring and interactive simulations compared to passive textbooks (19–21), the core advantage of our system lies in its immediate feedback mechanism. Other AI teaching tools mainly focus on case distribution or virtual patient dialogs (22, 23), but our platform directly uses the AI model to generate testing scenarios and instantly provides correct answers with visual annotations. Traditional static image learning often leads to memory decay. However, our platform’s immediate reinforcement loop helps students deeply internalize subtle features like MGO plugging. It should be noted that among the eight individual eyelid margin signs, only MGO plugging showed a statistically significant group difference in both score and accuracy rate. The remaining signs exhibited no significant between-group differences, which suggests that the benefit of the platform-based training might be most pronounced for signs with distinct, well-circumscribed morphological features. Although the absolute score difference of 3.58 points may appear modest, this 6.7% relative improvement in overall accuracy represents a meaningful educational gain within a short intervention period. Prior evidence suggests that even modest improvements in improvement can have substantial cumulative effects when scaled across a cohort of trainees.

The subgroup analysis revealed different educational benefits for medical students at varying academic stages. For undergraduate students, there was no significant difference in total scores, but the platform group had a significantly higher accuracy rate for rounding of posterior lid margin. Because undergraduates lack prior ophthalmological training, the high-frequency feedback from the AI system effectively helped them rapidly master fundamental and macroscopic morphological changes. Conversely, for graduate students, the platform group scored significantly higher in identifying MGO plugging in both score and accuracy. Since graduates already possessed a foundational understanding of ophthalmology, the system’s precise annotations helped them overcome learning bottlenecks associated with more detailed and specialized clinical signs.

Introducing this AI model into dry eye eyelid margin teaching may enhance student autonomy, potentially increase learning efficiency, and reduce memory forgetting. Moving forward, the platform can be further optimized in two main directions. First, AI algorithms can be further integrated into question bank construction, generation, and test feedback to set up personalized learning paths. Second, other AI models can be introduced to expand the platform to other dry eye-related signs. For example, integrating models for corneal nerves (24), conjunctival hyperemia (25), and meibomian gland identification (26) would help establish a comprehensive AI-assisted dry eye teaching system.

Despite these advantages, the present study has certain limitations. First, the sample size for the teaching evaluation was relatively small. Therefore, some comparisons that appeared to have differences did not reach statistical significance. The experiment was also conducted within a single medical institution. Second, this evaluation primarily focused on short-term learning outcomes, and long-term knowledge retention still requires subsequent follow-up tracking. Third, at a broader level, there is currently no standardized framework of competencies to facilitate the adoption of AI curricula in medical education (27), and faculty development in AI literacy remains underdeveloped (19, 28). Future studies should validate these findings across multiple centers with a broader and more diverse student population. Fourth, this study assessed only image-based diagnostic accuracy on a computer screen, not slit-lamp examination competency or real clinical performance, which are essential outcomes for clinical education. Fifth, the intervention and control conditions differed not only in the AI component but also in the mode of practice. Therefore, the observed benefit may partly reflect the effect of active practice and immediate feedback in general, rather than the AI component specifically. Future studies should compare the AI-based platform against an active non-AI practice condition using expert-provided feedback on the same image sets to isolate the unique contribution of the deep learning model.

In conclusion, this present study developed a teaching platform integrated with a deep learning model to teach lid margin abnormalities in patients with dry eye disease (DED). We enrolled students to compare the teaching results between the conventional and the platform learning method. The results suggest that compared with conventional teaching, using the platform for teaching and training can improve students’ overall accuracy in identifying abnormal eyelid margin signs. This improvement was particularly obvious in the identification of MGO plugging. It is important to emphasize that this study evaluated only short-term, image-based examination performance. Long-term knowledge retention, slit-lamp diagnostic ability, and real clinical performance were not assessed. This research provides a preliminary experience of integrating AI into teaching eyelid margin abnormalities. After further improvement, this platform could be used for teaching DED and even other ophthalmic imaging examinations that may support competency-based training in ophthalmology.

## Data Availability

The raw data supporting the conclusions of this article will be made available by the authors, without undue reservation.
