# Supplementary material for: Application of a deep learning-based system for eyelid margin signs identification training and testing in dry eye disease education
Source: Front Med (Lausanne). 2026 Jul 31;13:1896446. doi: 10.3389/fmed.2026.1896446 (PMC13474015; doi:10.3389/fmed.2026.1896446)
Supplement: Supplementary file 1 [file Supplementary_File_1.docx]

Figure S1: Participant flow diagram
